# When Myeloma Escapes the Bone Marrow: Extramedullary Disease in the Immunotherapy Era

**DOI:** 10.3390/cancers18152415

**Published:** 2026-07-27

**Authors:** Aimaz Afrough, Christen M. Dillard, Anne M. Alsup, Jimmy Lee, Samer Al Hadidi, Aishwarya Sannareddy, Pearl R. Abraham, Laura Turer, Danai Dima, Adeel M. Khan, Sean M. Taasan, Oren Pasvolsky, Krina K. Patel, Abdel Kareem Azab, Larry D. Anderson, Mahmoud R. Gaballa

**Affiliations:** 1 Myeloma, Waldenstrom’s, and Amyloidosis Program, Section of Hematologic Malignancies and Cellular Therapy, Simmons Comprehensive Cancer Center, UT Southwestern Medical Center, Dallas, TX 75390, USAlarry.anderson@utsouthwestern.edu (L.D.A.J.); 2 MD Anderson Cancer Center, Houston, TX 77030, USA; 3 Biomedical Engineering, UT Southwestern Medical Center, Dallas, TX 75390, USA; 4 Internal Medicine, UT Southwestern Medical Center, Dallas, TX 75390, USA; 5 Fred Hutchinson Cancer Center, Seattle, WA 98109, USA

**Keywords:** extramedullary myeloma, CNS myeloma, CAR T-cell therapy, bispecific antibodies, immunotherapy

## Abstract

Extramedullary disease (EMD)—the proliferation of clonal plasma cells in soft tissues without direct bone connection—represents one of the most challenging manifestations of multiple myeloma, associated with aggressive biology, treatment resistance, and poor outcomes. EMD is driven by distinct pathophysiologic mechanisms including downregulation of adhesion molecules, acquisition of high-risk cytogenetic abnormalities (del(17p), gain(1q)), activation of the RAS–MAPK pathway, epigenetic dysregulation such as EZH2 upregulation, and remodeling of the immune microenvironment toward an immunosuppressive, T-cell-depleted phenotype. Conventional therapies, including anti-CD38-based regimens, yield limited efficacy in EMD, with pooled overall response rates of approximately 20% in triple-class-exposed relapsed/refractory disease. T-cell-redirecting therapies have emerged as the most promising treatment strategy. Both chimeric antigen receptor (CAR) T-cell therapy and bispecific antibodies have demonstrated clinically meaningful activity in soft tissue EMD, with CAR T-cell therapy providing the deepest and most durable responses, and dual-targeting bispecific combinations showing particularly encouraging efficacy. Central nervous system (CNS) myeloma, the most devastating form of EMD, has historically carried a dismal prognosis. Emerging retrospective data suggest that both CAR T-cell therapy and bispecific antibodies can achieve meaningful CNS responses with acceptable safety profiles, as part of multimodal approaches incorporating CNS-directed therapies. Despite these advances, EMD remains associated with inferior outcomes even in the immunotherapy era, underscoring the need for strategies targeting the immunosuppressive microenvironment, novel therapeutic approaches, and prospective EMD-focused clinical trials. This review provides a comprehensive overview of the biology, classification, and evolving treatment landscape of both non-CNS and CNS EMD in the era of T-cell-redirecting immunotherapy.

## Simple Summary

Extramedullary disease (EMD) is a high-risk form of multiple myeloma in which myeloma cells spread outside the bone marrow into soft tissues or organs. Patients with EMD often have more aggressive disease, respond poorly to standard treatments, and experience worse outcomes. Research has identified several biologic changes that help myeloma cells survive outside the bone marrow and escape immune control. Recently, immune-based therapies such as CAR T-cell therapy and bispecific antibodies have shown promising activity in EMD, including some cases involving the central nervous system (CNS). Among these treatments, CAR T-cell therapy appears to produce the deepest and most durable responses. However, long-term disease control remains difficult, and EMD continues to be associated with worse survival even in the era of modern immunotherapy. This review summarizes the current understanding of EMD biology and discusses emerging treatment strategies for both non-CNS and CNS disease, highlighting the need for prospective clinical trials dedicated to this high-risk population.

## 1. Introduction

Multiple myeloma (MM) is a plasma cell malignancy characterized by clonal proliferation within the bone marrow (BM), leading to end-organ damage including bone destruction, cytopenias, renal dysfunction, and immune dysregulation [1,2].

Although traditionally considered a marrow-confined disease, plasma cells may extend beyond their native microenvironment, manifesting either as soft tissue plasmacytomas (STP) or, less commonly, as circulating disease in the form of plasma cell leukemia (PCL) [3].

Soft tissue involvement itself comprises biologically distinct patterns of spread. Paraskeletal disease (PSD), also referred to as paramedullary disease in some literature, arises when an osseous plasmacytoma breaches the cortical bone and extends directly into adjacent soft tissues, remaining anatomically contiguous with the underlying skeletal lesion. In contrast, extramedullary disease (EMD) refers to hematogenous dissemination of plasma cells to distant soft tissues without direct connection to bone [4].

Although the term ‘true EMD’ has been used in prior literature, EMD is defined by hematogenous dissemination independent of the bone marrow microenvironment, making the qualifier ‘true’ redundant. Accordingly, we use the term EMD throughout this review without additional qualifiers. Historically, paraskeletal and hematogenous lesions were often analyzed together, but accumulating evidence demonstrates meaningful differences in biology, prognosis, and therapeutic responsiveness, emphasizing the importance of precise classification in both clinical trials and real-world (RW) studies [4,5].

## 2. Classification and Epidemiology

EMD represents the overarching category of bone-independent plasma cell dissemination. Within this group, it can be subclassified as soft-tissue (non-central nervous system) EMD and central nervous system (CNS) EMD. Soft tissue (non-CNS) EMD most commonly involves soft tissues and organs such as the skin, skeletal muscle, liver, lymph nodes, pleura, etc. It may be present at diagnosis but is more frequently observed in the relapsed or refractory setting [5,6]. On the other hand, CNS EMD constitutes a distinct anatomic and clinical subset due to its unique therapeutic challenges and particularly poor prognosis and is most commonly observed in the relapsed setting. For conceptual and clinical clarity, we will discuss both in this review. These manifestations can present either at the time of diagnosis (primary) or at the time of relapse (secondary). The rate of EMD increases with relapse, with the incidence of EMD at diagnosis ranging from 1.7% to 4.5%, rising to 3.4% to 10% at relapse, while the frequency of PSD remains stable, ranging from 6% to 34.2% at both diagnosis and relapse [7,8]. A higher incidence of EMD has been reported in recent decades. However, a retrospective study [9] analyzing 1003 patients diagnosed between 1971 and 2007, which included both EMD and PSD plasmacytomas under a broader definition of EMD, found that the observed increase was not influenced by prior exposure to high-dose chemotherapy or stem cell transplantation.

This rise in EMD is largely attributed to improved detection through systematic use of PET/CT and to the extended survival of MM patients under successive lines of therapy, which increases cumulative exposure to selective therapeutic pressure and the opportunity for clonal evolution toward marrow-independent growth [7,9,10].

## 3. Pathophysiology of EMD

**Figure 1:**
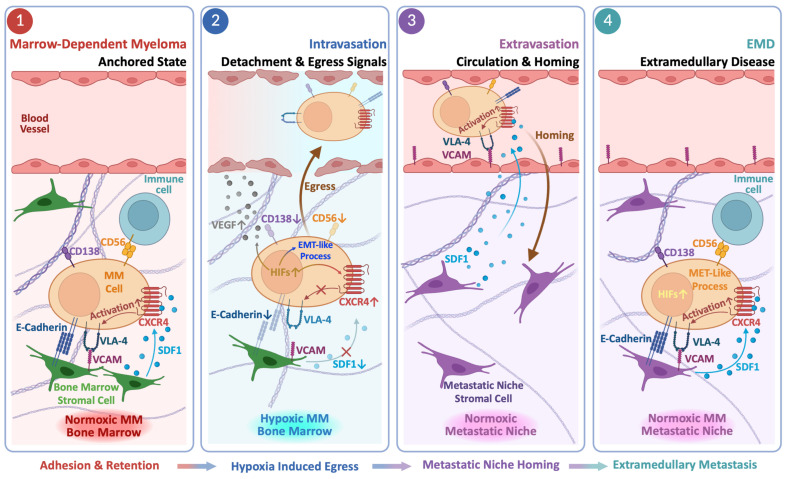
Proposed model of multiple myeloma dissemination from bone marrow to EMD. (**1**) In the normoxic bone marrow (BM), MM cells are retained through CD138, CD56, E-cadherin, VLA-4/VCAM-1, and CXCR4/SDF-1 interactions. (**2**) Hypoxia induces HIF-mediated EMT-like changes that reduce adhesion, increase migration, and promote BM egress. (**3**) Circulating MM cells home to distant niches through SDF-1 gradients and VLA-4/VCAM-1 interactions. (**4**) At extramedullary sites, MM cells acquire relative microenvironmental independence and undergo MET-like adaptation, supporting tumor growth. Together, hypoxia, adhesion signaling, and niche adaptation drive MM dissemination and progression to EMD. Created with BioRender.com.

Myeloma progression is increasingly recognized as a dynamic process involving sequential transitions between a BM-dependent state and disseminated disease [11,12,13,14,15,16,17,18,19,20,21,22,23,24]. This section summarizes a conceptual model of MM dissemination, highlighting the molecular and microenvironmental mechanisms that govern retention within BM, intravasation into the circulation, homing to distant sites, and eventual development of EMD (Figure 1).

### 3.1. Bone Marrow-Dependent Anchored State

MM cells are highly dependent on the BM microenvironment for survival, proliferation, and drug resistance [12,13,15,25,26,27,28]. Retention within the BM is maintained through multiple adhesion and chemokine-mediated interactions. The CXCR4/SDF-1 axis serves as a central homing and retention mechanism, where stromal-derived factor-1 (SDF-1/CXCL12) secreted by BM stromal cells attracts CXCR4-expressing MM cells. Concurrently, adhesion molecules including VLA-4/VCAM-1, CD138, CD56, and E-cadherin reinforce physical attachment to stromal cells and extracellular matrix components. These interactions activate pro-survival signaling pathways and establish a relatively stable, normoxic niche that favors tumor growth while limiting dissemination. At this stage, MM cells exhibit a highly adhesive phenotype characterized by strong interactions with both stromal and endothelial compartments.

Disrupting these retention mechanisms has raised concerns that MM cell mobilization could promote EMD. The CXCR4 antagonist plerixafor is widely used for stem cell mobilization. A phase I/II trial evaluating plerixafor in combination with bortezomib, as a chemosensitization strategy in RRMM, directly addressed this concern and showed no evidence that plerixafor-induced mobilization promoted EMD development [29]. However, the study had a relatively short follow-up, which limits definitive conclusions regarding long-term EMD risk. Similarly, bortezomib downregulates VLA-4 expression on MM cells, disrupting VLA-4/VCAM-1-mediated BM adhesion and overcoming cell adhesion-mediated drug resistance [30]. In a murine model, CRISPR-mediated ablation of VLA-4 in myeloma cells redirected tumor spread from medullary to extramedullary sites [31], raising the theoretical concern that therapeutic VLA-4 disruption could facilitate dissemination beyond the BM. However, clinical studies have not demonstrated increased EMD incidence with bortezomib-based regimens [32].

### 3.2. Hypoxia-Induced Egress and Intravasation

Disease progression and increasing tumor burden generate localized hypoxia within the BM [33]. Hypoxia-inducible factors (HIFs) orchestrate a transcriptional program that promotes MM cell detachment and migration, and stem-cell-like properties [19]. This transition resembles a partial epithelial-to-mesenchymal transition (EMT-like process), although plasma cells are not epithelial in origin. Hypoxia is associated with downregulation of adhesion molecules such as CD138, CD56, and E-cadherin, reducing interactions with stromal elements [19,21,33]. Simultaneously, CXCR4 expression increases, enhancing migratory responsiveness, while alterations in VLA-4 activity weaken BM retention, due to reduced CXCR4 activation by reduced SDF-1 secretion from the stroma [11,33]. Reduced local SDF-1 gradients and increased angiogenic signaling, including VEGF production, further facilitate movement toward the vascular compartment [11,13,14,33]. Collectively, these changes shift MM cells from a sessile, BM-dependent phenotype toward a motile and invasive state capable of intravasation and systemic dissemination.

### 3.3. Circulation and Homing to Secondary Niches

Following entry into the circulation, disseminated MM cells encounter distant microenvironments that may support metastatic colonization. Successful homing requires re-engagement of adhesive and chemotactic pathways; gradients of SDF-1 produced within permissive niches attract circulating CXCR4-high MM cells, while activation of VLA-4 enables interaction with VCAM-1-expressing stromal and endothelial cells. This process mirrors leukocyte trafficking, involving sequential adhesion, transmigration, and tissue colonization [11,12,13,33]. Once localized to a new niche, MM cells begin re-establishing interactions with the surrounding microenvironment, allowing survival and expansion at metastatic sites. These secondary niches may reside within distant locations.

### 3.4. Extramedullary Disease and Microenvironmental Independence

The final stage of dissemination is the emergence of EMD characterized by growth outside the BM. Unlike BM-confined MM, EMD cells exhibit reduced dependence on stromal support and acquire features resembling a mesenchymal-to-epithelial transition (MET-like process), allowing adaptation to non-BM tissues [33].

Although CXCR4 signaling and integrin-mediated interactions remain important, EMD cells become progressively less reliant on classical BM retention mechanisms. The transition from BM-dependent disease to EMD therefore reflects a continuum of evolving tumor–microenvironment interactions, driven by hypoxia, loss of adhesion, chemokine signaling, and niche adaptation. Understanding these mechanisms provides a framework for identifying therapeutic strategies aimed at preventing dissemination, disrupting metastatic homing, and treating extramedullary progression in MM. Mechanistically, EMD cells compensate for the loss of BM-derived survival signals through autocrine and paracrine growth factor loops, and constitutive activation of proliferative pathways such as MAPK [34] (discussed in detail in Section 4.1).

## 4. Genomic Landscape of EMD

### 4.1. Genomic Architecture and Cooperating Alterations

EMD is characterized by significantly greater genomic complexity than BM-confined myeloma. Transcriptomic analyses of soft tissue EMD samples identified a frequent co-occurrence of 1q21 gain/amplification and MAPK pathway mutations, which was associated with a significantly increased risk of EMD development. Validation using the CoMMpass dataset (*n* = 699; NCT01454297) confirmed this association, demonstrating that the combination of *KRAS* mutations and 1q21 gain/amplification—rather than either alteration alone—was independently associated with a higher risk of developing EMD in multivariable analysis (HR = 2.4, *p* = 0.011) [35]. Independently, whole exome sequencing of EMD tumor tissue identified MAPK pathway mutations in 94% of EMD samples versus 60% of matched BM aspirates, with frequent clonal alterations in *NRAS*, *KRAS*, and *BRAF*, supporting their central role in EMD pathogenesis [36]. Critically, *NRAS*, *KRAS*, and *BRAF* mutations were clonal in approximately 50% of EMD cases, suggesting these represent founding, disease-driving events rather than subclonal bystanders, strengthening the therapeutic rationale for MAPK pathway targeting in this setting [36].

Most myeloma cells at diagnosis depend on the BM microenvironment for survival signals, largely mediated through stromal IL-6/STAT3 signaling. Activating *KRAS*, *NRAS*, or *BRAF* mutations constitutively activate the RAS–MAPK pathway, providing cell-intrinsic proliferative signaling that reduces dependence on the BM microenvironment [37]. Concurrently, IL6R is located within the 1q21 locus, and 1q21 gain/amplification confers IL-6 hypersensitivity through IL6R overexpression and STAT3 hyperactivation, alongside anti-apoptotic resistance through MCL1 and CKS1B amplification [38]. This IL-6 hypersensitivity is further amplified by the VEGF/IL-6 paracrine loop, in which VEGF secreted by myeloma cells stimulates IL-6 production from stromal and endothelial cells (14–27-fold increase), while IL-6 in turn upregulates VEGF expression in myeloma cells, creating a self-reinforcing amplification circuit [39]. Clinically, serum VEGF levels are significantly elevated in patients with EMD and predict poor chemotherapy response [40]. Single-cell RNA sequencing from 15 paired marrow and EMD samples demonstrated higher expression of IL-6, CCL3, and other growth factors in EMD cells compared to both primary and refractory BM cells [34], and IL6R gains were observed in 60% of EMD samples [41]. Collectively, the combination of constitutive MAPK-driven proliferation, 1q21-mediated IL-6 hypersensitivity, VEGF/IL-6 paracrine amplification, and apoptotic resistance equips myeloma cells for BM-independent survival and EMD progression.

Notably, the prognostic significance of RAS/MAPK mutations in myeloma remains under evaluation. In the IRMMa risk-prognostic model, which integrated clinical, genomic, and treatment variables from 1933 BM-derived NDMM samples, *RAS* pathway mutations did not significantly influence overall survival (OS) and were therefore not included among the prognostic features, despite being the most frequently mutated driver genes in the cohort [*KRAS* (24.3%), *NRAS* (20.1%), *BRAF* (7.8%)] [42]. This contrasts with studies using EMD tissue, where RAS/MAPK mutations are clonal, present at markedly higher frequency, and appear to play a distinct pathogenic role in enabling microenvironment-independent survival [36,41].

However, MAPK pathway activation alone appears insufficient for EMD development. Additional cooperating genomic events are enriched in EMD and likely contribute to disease progression [43]. A comprehensive meta-analysis of 41 studies (9424 patients) characterizing the cytogenetic landscape of EMD confirmed higher frequencies of del(17p)/TP53 deletion and del(13q)/RB1 deletion, and lower frequencies of hyperdiploidy and t(11;14), compared to patients without EMD [44].

In an analysis of 528 molecularly tested MM patients (2014–2021), the poor outcomes associated with 1q21 gain and del(17p) were largely attributable to EMD development. The same study demonstrated that not all *TP53* aberrations contribute equally to EMD: non-EMD patients harbored mutations distributed throughout the gene, while EMD patients showed enrichment of mutations in the C-terminus, including *TP53* gain-of-function mutations, though the functional significance of this finding in myeloma remains to be determined [43].

Beyond *TP53*, biallelic inactivation of *MAX* (MYC-associated factor X), a critical heterodimerization partner of MYC, further contributes to EMD pathogenesis. *MAX* biallelic loss was identified in 22% of EMD samples and was exclusively clonal, suggesting a selective growth advantage. *MAX* loss deregulates the MYC transcriptional program, and small-molecule inhibitors of MYC-MAX interactions represent an emerging therapeutic avenue worthy of investigation in this subgroup [36].

Through whole-genome sequencing of EMD samples, two distinct molecular subtypes have been identified: a MAPK-driven group (approximately 80%), characterized by clonal *RAS*/*BRAF* codon 61 mutations and the SBS9 mutational signature, and a hypermutated group (approximately 20%), driven by apolipoprotein B mRNA-editing catalytic polypeptide (APOBEC) mutagenesis (SBS2/SBS13)—a family of DNA editing enzymes that deaminate cytosine [45,46]—which is associated with MAF/MAFB translocations [t(14;16)/t(14;20)] [41]. Notably, highly expressed APOBEC3B, which is independently associated with poor prognosis in MM, constitutively generates genomic instability, with mutations observed in endogenous genes frequently mutated in myeloma, including *TP53* [47]. These subtypes had meaningfully different clinical trajectories: long-term survivors beyond 4 years from EMD diagnosis were exclusively in the MAPK-driven group, suggesting that mutational signature analysis may inform risk stratification and, ultimately, therapeutic selection in EMD [41].

### 4.2. Therapeutic Target Expression and Immune Evasion

Beyond structural genomic alterations, transcriptomic analyses have revealed clinically relevant changes in therapeutic target expression and immune evasion mechanisms in EMD. A comparison of EMD tissue (*n* = 14), BM from newly diagnosed MM (NDMM) (*n* = 14), and BM from relapsed refractory MM (RRMM) without EMD (*n* = 14) [35] demonstrated decreased expression of CD38, SLAMF7, G protein-coupled receptor, class C, group 5, member D (GPRC5D), and FCRH5 in EMD cells, with significant upregulation of EZH2 and CD70. Notably, B-cell maturation antigen (BCMA) expression was not deregulated, suggesting that BCMA-targeting therapies may retain activity against EMD. Spatial transcriptomics of 14 EMD biopsies further confirmed heterogeneous expression of GPRC5D and BCMA within individual EMD lesions, supporting the rationale for dual- or multi-target strategies [48].

EZH2, an epigenetic silencer of tumor suppressor genes not expressed in normal BM plasma cells [49], becomes upregulated during myeloma progression and is associated with shorter progression-free survival (PFS) [50]. EZH2 promotes myeloma cell proliferation [51], and drives immune evasion by impairing NK and T-cell recruitment through suppression of CXCL10 (an NK/T-cell-recruiting chemokine) [52], while stabilizing FOXP3 (master transcriptional regulator of regulatory T-cell (Treg)) to maintain their immunosuppressive function [53] and inducing myeloid-derived suppressor cell (MDSC) activity [53]—mechanisms particularly relevant in the immune-depleted EMD niche. In an in vitro study, tazemetostat—the only FDA-approved EZH2 inhibitor (approved for follicular lymphoma [54]—in combination with immunomodulatory drugs (IMiDs) or cereblon E3 ligase modulators (CELMoDs), produced synergistic cell death in IMiD-resistant myeloma cell lines through a cereblon-dependent mechanism that enhances IRF4 suppression [55]. Given that EZH2 is significantly upregulated in EMD cells [35], its dual role in promoting proliferation and immune evasion makes it a particularly attractive therapeutic target in this setting.

At the tissue level, EMD cells persistently express class I MHC molecules and upregulate inhibitory ligands for both cytotoxic T cells and NK cells, while infiltrating CTLs and NK cells show gene-expression profiles indicative of functional compromise [34]. Complementing these target-expression findings, spatial transcriptomic analyses have demonstrated functionally exhausted TIM-3+/PD-1+ T-cells diffusely colocalized with myeloma cells within tumor masses, whereas functional, activated CD8+ T-cells and M1 macrophages were confined to tumor-free regions at biopsy margins [48]. These findings underscore the profoundly immunosuppressive nature of the EMD niche and provide a rationale for strategies aimed at restoring T-cell competence in this compartment.

CD70 (TNFSF7), a member of the tumor necrosis factor receptor superfamily [56], is progressively upregulated with myeloma stage and reaches its highest levels in EMD. CD70/CD27 signaling activates MAPK/ERK and Wnt/β-catenin pathways in myeloma xenograft models [57], and chronic CD70/CD27 interaction promotes T-cell exhaustion and apoptosis, contributing to immune evasion [58]. CD70 expression is also regulated by HIFs [58], linking it to hypoxia-driven dissemination. Preclinically, an ADCC-enhanced anti-CD70 antibody (cusatuzumab) combined with NK cells significantly suppressed EMD tumor progression [57]. Independently, an optimized CD27-based anti-CD70 CAR T-cell construct achieved >80-fold improved expansion over conventional scFv-based designs in vivo [59]. No clinical trials of anti-CD70 therapies in myeloma have been initiated, but CD70 upregulation in EMD [35,57], its recognition as a favorable immunotherapy target in other malignancies, and the feasibility of dual-targeting CAR T-cell constructs against both CD70 and BCMA [59] position CD70 as a particularly attractive therapeutic target for EMD.

## 5. Treatment Strategies

The presence of EMD remains a well-established marker of inferior prognosis across all stages of disease. In transplant-eligible patients with NDMM, high-dose chemotherapy followed by autologous stem cell transplantation (ASCT) remains a cornerstone of management for those with EMD who achieve a response to induction therapy. A large EBMT registry analysis of 3744 NDMM patients demonstrated that upfront ASCT can yield comparable PFS in patients with single-site EMD versus those without EMD; however, patients with multiple sites of organ involvement had significantly worse outcomes [60].

In the relapsed refractory setting, outcomes for patients with EMD treated with conventional regimens remain poor. A retrospective study on daratumumab demonstrated that patients with any STP (including EMD and PSD) had significantly shorter median PFS (1.4 vs. 6.2 months; *p* = 0.002) and OS (4.6 vs. 15.4 months; *p* = 0.042) compared to patients without STP [61].

Of note, thalidomide, the first-generation IMiD, showed no meaningful activity against STP, with a 0% response rate compared to 53–59% in patients without STP involvement [62,63], likely reflecting the dependence of thalidomide’s anti-myeloma mechanism on the BM microenvironment [64].

A pooled analysis of the LocoMMotion and MoMMent-1 RW cohorts (2019–2022) evaluated outcomes in 302 patients, including 29 with STP (15 EMD and 14 PSD) [65]. Patients with STP demonstrated markedly inferior outcomes, with an overall response rate (ORR) of 24.1%, median PFS of 2.66 vs. 5.09 months, and median OS of 7.16 vs. 15.51 months. The 6- and 12-month PFS rates were 20.7% and 15.5%, respectively. Notably, 21 different standard-of-care (SOC) regimens were used across the cohort, including proteasome inhibitor (PI)-based, IMiD-based, anti-CD38 monoclonal antibody-based, and chemotherapy-based combinations. Of note, BCMA-targeted therapy was administered in only 7% (*n* = 2; belantamab mafodotin). None of the patients received CAR T-cell therapy or bispecific antibodies (BsAb), underscoring the limited exposure of this high-risk population to modern T-cell-redirecting therapies during that timeframe. Furthermore, a meta-analysis of standard regimens (including anti-CD38-based therapies) in patients with triple-class-exposed RRMM across clinical trial studies from 2002 to 2024 demonstrated that the pooled ORR for patients with EMD was 20.7% compared with 66.2% in those without EMD, with shorter median PFS (6.3 vs. 12.9 months) and OS (21.0 vs. 39.0 months) [66].

Nonetheless, the limited efficacy of conventional regimens in patients with EMD who are not transplant-eligible, who relapse after ASCT, or who are refractory to standard therapies underscores the need for T-cell-redirecting therapies—the subject of the following sections. The summary is presented in Table 1.

### 5.1. Antibody–Drug Conjugates (ADCs)

BCMA antibody–drug conjugates (ADCs) have demonstrated limited efficacy in patients with RRMM and STP. In DREAMM-2 [67,68], among patients treated with single-agent belantamab mafodotin (2.5 mg/kg), 23% (22/97) had STP (including EMD or PSD). Only one patient (4.5%) responded, while 36% had stable disease. Median PFS was 1.1 months, and median OS was 13.4 months, highlighting the poor outcomes associated with belantamab mafodotin in this high-risk population. A RW analysis of belantamab mafodotin monotherapy (2019–2023; median follow-up 11.3 months) showed significantly poorer outcomes in patients with EMD, with median PFS of 2 vs. 10 months and median OS of 5 vs. 22 months compared with those without EMD [69].

### 5.2. T-Cell Engager (TCE) Antibodies

#### 5.2.1. Bispecific Antibodies

##### BCMA-Directed Bispecific Antibody

The MajesTEC-1 trial, which led to the approval of teclistamab, the first BCMA-directed BsAb, defined EMD strictly by the presence of EMD, without bone-based plasmacytoma (PSD). In this trial, 17% (*n* = 28) of patients met this criterion, and these patients demonstrated a lower ORR of approximately 35%, compared to 63.0% in the overall population [70].

Similarly, the LINKER-MM1 trial, which led to the approval of linvoseltamab in the RRMM setting, included 16% of patients (*n* = 19) with EMD (≥2 cm), reporting an ORR of 52.6% vs. 70.9% in all patients at 200 mg full dose [71].

In the MagnetisMM-3 trial, elranatamab, another BCMA-BsAb, employed a broad definition of STP, encompassing both EMD and PSD lesions. The ORR was 38.5% among patients with STP vs. 71.4% in those without STP. Notably, despite the lower initial ORR in patients with STP, the duration of response was comparable between groups (77.9% vs. 70.6%) [72].

In our prior RW analysis evaluating teclistamab, EMD was associated with significantly inferior outcomes. The ORR was 38% in patients with EMD, compared with 54% in those with PSD and 62.4% in patients without STP. A similar pattern was observed for survival outcomes, with median PFS of 1.4 months in the EMD group, 6.5 months in the PSD group, and 8.95 months among patients without STP. Median OS was 9.54 months in patients with EMD, whereas it was not reached in either the PSD or no-STP groups. On multivariable analysis, EMD remained an independent adverse prognostic factor for both PFS and OS [73].

In RW analyses of elranatamab, elevated LDH independently predicted inferior PFS and OS, whereas EMD was not independently prognostic. Although ferritin was excluded from multivariable modeling because of missing data, restricted cubic spline analyses demonstrated a progressively increasing risk of progression and death with higher LDH and ferritin levels, underscoring the impact of systemic tumor burden and inflammatory activation on outcomes [74]. In another study, comparison with RW IMWG SOC in triple-class-exposed RRMM showed a trend toward better PFS with linvoseltamab (HR 0.30, 95% CI 0.09–1.00) [75].

No head-to-head randomized trials have compared the approved BCMA-directed BsAb. Matching-adjusted indirect comparisons (MAICs) suggest potential efficacy differences, but these findings remain hypothesis-generating. In one comparison, elranatamab showed higher adjusted ORR (75.3% vs. 63.0%) and longer PFS (Hazard ratio (HR) 0.59) vs teclistamab [76]. Linvoseltamab demonstrated longer duration of response (DOR) (HR 0.54), PFS (HR 0.55), and OS (HR 0.64) vs teclistamab [77], and higher ORR (71.5% vs. 61.0%) and ≥CR (50.5% vs. 37.4%), with OS favoring linvoseltamab by restricted mean survival time analysis (difference: 3.47 months; *p* = 0.04), though conventional PFS and DOR differences were not statistically significant [78].

##### GPRC5D-Directed Bispecific Antibody

The phase 1, MonumenTAL-1 trial led to the approval of talquetamab. Among patients treated with subcutaneous talquetamab at 0.4 mg/kg weekly, the ORR was 45.5% in patients with EMD compared with 70% in the overall study population. Similarly, in the 0.8 mg/kg every-2-week cohort, the ORR was 40% in patients with EMD vs. 64% in the overall population [79]. In a post hoc analysis of phase 1/2 with a median follow-up of 25.6 months in the 0.4 mg/kg cohort and 19.4 months in the 0.8 mg/kg cohort, the presence of EMD was associated with markedly inferior PFS. In the 0.4 mg/kg weekly cohort, median PFS was 4.6 months (95% CI, 2.8–5.6) in patients with EMD compared with 9.2 months (95% CI, 7.0–11.8) in those without EMD. A similar pattern was observed in the 0.8 mg/kg every-2-week cohort, where median PFS was 3.4 months (95% CI, 2.1–5.4) in patients with EMD versus 16.9 months (95% CI, 11.3–not estimable [NE]) in those without EMD [80].

A multicenter RW study of 360 patients treated with talquetamab across 15 U.S. centers, with median follow-up of 12.8 months, demonstrated preserved response rates despite inferior survival outcomes in patients with STP [81]. Among 97 patients with EMD (27%), 22 with PSD (6%), and 241 without STP (67%), ORRs were similar across groups (68%, 63%, and 65%, respectively; *p* = 0.8). However, median PFS was significantly shorter in patients with EMD and PSD compared with those without STP (4.3, 4.5, and 7.8 months, respectively; *p* = 0.009), while median OS also trended shorter (10.3, 13.0 months, and not reached; *p* = 0.070). Although EMD was associated with inferior PFS and OS on univariable analysis, it was not independently associated with survival after adjustment for clinical and laboratory factors. Elevated LDH and ferritin independently predicted inferior PFS, with LDH also associated with worse OS. Overall, these findings suggest that talquetamab retains meaningful activity in EMD, although responses remain less durable in this high-risk population.

In a separate comparative analysis evaluating the efficacy of talquetamab against RW physician’s choice in triple-class-exposed patients, patients were divided into three cohorts of T-cell redirecting therapies (TRT), including CAR T-cell or BsAb: those who received subcutaneous talquetamab 0.4 mg/kg weekly (QW; *n* = 143), 0.8 mg/kg every 2-week (Q2W; *n* = 154), and a cohort with prior BCMA-directed TRT therapy (*n* = 75). This analysis demonstrated improved efficacy and survival outcomes with talquetamab compared with RW physician’s choice, regardless of prior BCMA-directed TRT. In TRT-naïve patients, talquetamab was associated with a 53% reduction in the risk of progression and a 65% reduction in the risk of death, while in patients with prior BCMA-directed TRT, the corresponding risk reductions were 70% and 63%, respectively, underscoring consistent benefit across subgroups [82].

##### Combination of Bispecific Antibodies with Different Targets

As monotherapy with T-cell-redirecting BsAb generally yields modest responses in EMD (ORR 35–45%) [70,73,79,83], the phase 1b RedirecTT-1 trial evaluated a combination approach using the BCMA-directed BsAb, teclistamab, and the GPRC5D-directed BsAb, talquetamab, in patients with RRMM. Among treated patients, 36% (34/94) across all dose levels and 41% (18/44) at the recommended phase 2 dose (RP2D) had at least one EMD lesion ≥2 cm. At RP2D, the trial reported an ORR of 61% in patients with EMD compared with 80% in all patients, with 18-month PFS rates of 53% vs. 70% [84]. In a subsequent dedicated phase 2 study enrolling exclusively patients with EMD (*n* = 90; median of 4 prior lines of therapy; 20% prior BCMA CAR T-cell therapy; 84.4% triple-class refractory; 35.6% penta-refractory; 12% prior belantamab mafodotin exposure; and 9% prior FcRH5-directed bispecific exposure; 0% of the patients had prior exposure to BCMA- or GPRC5D-directed BsAb), the ORR was 79%, with 54% achieving ≥CR. At a median follow-up of 12.6 months, the 12-month PFS was 61% (95% CI, 50–71), and 12-month OS was 74% (95% CI, 63–83) [85]. Despite these promising results, the study excluded patients with CNS involvement and therefore cannot address outcomes in this important subgroup. Additionally, treatment-related deaths were reported in 10 patients, including five due to infection, which raises concern regarding real-world implementation [86]; further RW data are needed to determine the full scope of its applicability. On the other hand, the dual-targeting strategy is particularly promising given the spatial genomic heterogeneity of MM. Multi-region sequencing studies have demonstrated that MM evolves through ongoing clonal competition and site-specific driver alterations [87]. In parallel, spatial transcriptomic analyses have revealed heterogeneous BCMA and GPRC5D expression within individual EMD lesions [48], further supporting the rationale for simultaneous multi-epitope targeting.

#### 5.2.2. Trispecific Antibodies

Early-phase clinical data for trispecific antibodies, including ramantamig (BCMA × GPRC5D × CD3) [88], and ISB 2001 (BCMA × CD38 × CD3) [89], have shown promising overall response rates; however, EMD-specific subgroup analyses remain unavailable across trispecific trials. Considering the heterogeneous and frequently downregulated expression of therapeutic targets in EMD cells [35], trispecific constructs incorporating dual-antigen targeting may offer a particularly rational therapeutic approach. Prospective EMD-specific reporting should therefore be prioritized in ongoing and future trispecific studies.

### 5.3. CAR T-Cell Therapy

#### 5.3.1. BCMA-Directed CAR T-Cell Therapy

BCMA-directed CAR T-cell therapies demonstrate high response rates in heavily pretreated myeloma; however, outcomes remain inferior in patients with EMD involvement. In phase 2 of the KarMMa trial, which examined idecabtagene vicleucel (ide-cel) in RRMM at the 4th or later line of therapy, STP were reported in 39% of enrolled patients. Notably, the trial used a broad definition of EMD that included both EMD and PSD. The response rates in this subgroup remained robust, with an ORR of 70% in comparison to 73% in all patients [90].

Similarly, the phase Ib/II CARTITUDE-1 trial [91] evaluated ciltacabtagene autoleucel (cilta-cel) in patients treated beyond the 4th line of therapy and included 19 patients with STP (EMD+ PSD). Although responses were universal in this subgroup (ORR 100%, 95% CI: 82.4–100), durability of response appeared shorter compared with the overall study population, with a median DOR of 12.9 months (95% CI: 3.5–NE) and a median PFS of 13.8 months (95% CI: 5.3–NE), whereas these endpoints were not reached (NR) in the overall cohort. With extended follow-up at 27 months, the PFS rate was 47.4% in patients with STP compared with 54.9% in the overall cohort, while the corresponding OS rates were 52.1% and 70.4%, respectively [91]. Long-term follow-up [92] at 61.3 months demonstrated durable responses, with approximately one-third of patients remaining treatment-free and progression-free. The proportion of patients with EMD was similar among those who remained progression-free compared with those who experienced disease progression within five years after treatment.

RW data have further characterized outcomes in EMD, particularly when defined more strictly by excluding PSD lesions [93,94]. In one multicenter RW analysis including 152 patients treated with ide-cel or cilta-cel beyond the fourth line of therapy, patients with EMD (*n* = 47) experienced significantly worse outcomes compared with those without EMD (*n* = 105), with shorter median PFS (5.1 vs. 12.4 months; *p* < 0.0001) and OS (12.2 vs. 27.5 months; *p* = 0.00058) [93].

Consistent observations were reported in another multicenter study evaluating 351 patients treated with ide-cel beyond the fourth line of therapy, in which individuals with EMD (*n* = 84) had inferior PFS (5.3 vs. 11.1 months; *p* < 0.0001) and OS (14.8 vs. 26.9 months; *p* = 0.006) compared with those without EMD (*n* = 267) [94]. Together, these studies highlight the persistent adverse prognostic impact of EMD even in the era of CAR T-cell therapy.

Additional RW evidence from patients receiving cilta-cel beyond the fourth line of therapy also identified EMD as an independent adverse prognostic factor. In this cohort, 26% of patients (*n* = 60) had EMD and demonstrated a significantly shorter PFS. Multivariable analysis further confirmed the association between EMD and inferior survival outcomes, with HR of 1.96 for PFS (95% CI: 1.19–3.23, *p* = 0.009) and 1.88 for OS (95% CI: 1.04–3.42, *p* = 0.04) [95].

Investigators have explored the use of CAR T-cell therapy earlier in the disease course, when T-cell fitness may be greater and overall tumor burden lower. The phase III KarMMa-3 trial [96] evaluated ide-cel in patients who had received 2–4 prior lines of therapy and had refractory disease, comparing the treatment with SOC regimens. Among patients with STP (EMD + PSD), ide-cel demonstrated improved PFS compared with SOC, with median PFS of 7.2 months (95% CI: 4.0–11.8) vs. 2.0 months (95% CI: 1.3–3.0).

A similar pattern was observed in the phase III CARTITUDE-4 trial, which investigated cilta-cel in patients who had received one to three prior lines of therapy and were refractory to lenalidomide [97]. At a median follow-up of 33.6 months, median PFS was shorter among patients with STP (EMD + PSD) compared with those without plasmacytomas (16.9 months [95% CI: 3.6-NE] vs. NE [95% CI: 37.1-NE] [98].

Direct comparisons between clinical trials and RW studies remain challenging due to differences in patient populations, definitions of EMD, study design, and the frequency of imaging, which is typically less standardized and less frequent in RW practice compared with clinical trials. Notably, extended follow-up of CARTITUDE-1 demonstrated that durable long-term disease control after cilta-cel therapy can occur irrespective of baseline EMD status. Nevertheless, the improved outcomes observed in CARTITUDE-4 suggest that earlier integration of cellular therapies may further mitigate the adverse prognostic impact associated with EMD in MM. That being said, the recent RW registry analysis of cilta-cel in early lines (1–3) (*n* = 177) vs. late lines (>3) (*n* = 429) from the German national registry (DRST) showed that EMD was an independent adverse prognostic factor in both early and late line cilta-cel. Notably, while high-risk cytogenetics were not associated with inferior PFS in the early group, EMD remained adversely prognostic regardless of treatment line, underscoring the distinct biological challenge posed by EMD [99].

A multicenter retrospective study of 80 patients with EMD demonstrated superior outcomes with CAR T-cell therapy compared with BsAb, including higher ORR (82% ide-cel; 100% cilta-cel vs. 36% teclistamab; 29% talquetamab), higher complete EMD resolution rates (41–50% vs. 24–18%), and longer median PFS (7.3 months—NR vs. 4.0–2.6 months). Successful debulking therapy before CAR T-cell infusion (partial response or better) was associated with prolonged PFS. Time to EMD recurrence was also longer after CAR T-cell therapy (NR with cilta-cel and 8.5 months with ide-cel). Relapse patterns also differed markedly: 35% of patients progressing after CAR T-cell therapy had serologic-only progression while maintaining EMD remission, whereas 95% of progressions after BsAb therapy involved EMD recurrence [100].

#### 5.3.2. GPRC5D-Directed CAR T Therapy

GPRC5D-targeted CAR T-cell therapy is also under evaluation. The phase 1 study of MCARH109 (GPRC5D-directed CAR T-cells) in RRMM reported an ORR of 71% (12/17) across the entire cohort, including responses in patients with biopsy-proven EMD (5/8, 62.5%) [101]. At a median follow-up of 37 months, the overall median duration of response was 8.6 months (95% CI, 5.7 to NR), with two patients sustaining stringent complete responses at 32 and 41 months. In the EMD subgroup, the duration of response among the 5 responders ranged from 3.4 to 26.1 months (median 5.7 months), based on supplementary data from the updated analysis. Possible GPRC5D loss by immunohistochemistry was observed in 60% (6/10) of patients at relapse [102].

Another GPRC5D-targeted CAR T-cell therapy, arlocabtagene autoleucel (arlo-cel), was also recently reported to have strong activity in 31 out of 36 (86%) of those with EMD, similar to the 87% ORR in the overall study (*n* = 84) in a population that had received at least 3 prior LOT and 49% had prior BCMA-directed therapy exposure [103].

#### 5.3.3. Dual-Targeted CAR T Therapy

A phase 1 study evaluated dual-targeted BCMA/GPRC5D CAR T-cell therapy specifically in patients with EMD in the RRMM setting (*n* = 9 evaluable). The ORR was 100%, with 44.4% achieving CR or better. At a median follow-up of 6.08 months, the 1-year PFS and OS rates were 63% and 60%, respectively. The safety profile was favorable, with only grade 1–2 cytokine release syndrome (CRS) and no immune effector cell-associated neurotoxicity syndrome (ICANS) observed [104].

**Table 1:** Efficacy of bispecific antibodies, CAR T-cell therapies, and antibody–drug conjugates in multiple myeloma with extramedullary involvement: clinical trial and real-world data.

Trial/Study	Therapy	Phase	EMD Definition	N (EMD/Total)	ORR EMD	ORR Comparator ^†^	mPFS EMD	mPFS Comparator ^†^	mOS EMD	mOS Comparator ^†^	Median Follow Up	Ref ^Δ^
**ADC**												
DREAMM-2	Belantamab mafodotin (2.5 mg/Kg Q3W)	Ph2	STP (EMD + PSK)	22/97	4.5%	32% (all)	1.1 mo	2.8 mo (all)	13.4 mo	15.3 mo (all)	12.5 mo	[67,68]
**BCMA Bispecific Antibodies**												
MajesTEC-1	Teclistamab (1.5 mg QW)	Ph1-2	EMD	28/165	35.7%	63.0% (all)	NRep	11.3 mo (all)	NRep	18.3 mo (all)	14.1 mo	[70]
Riedhammer et al.	Teclistamab	RWE	EMD	43/119	37.0%	59.3% (all)	2.1 mo	8.7 mo (all)	NRep	NR (all)	5.5 mo	[83]
Afrough et al.	Teclistamab	RWE	EMD	109/358	38.0%	62.4% (no-STP)	1.4 mo	8.95 mo (no-STP)	9.54 mo	NR (no STP)	9.9 mo	[73]
MagnetisMM-3	Elranatamab	Ph2	STP (EMD + PSD)	39/123	38.5%	71.4% (no-STP)	NRep	NRep	NRep	NRep	14.7 mo	[72]
LINKER-MM1	Linvoseltamab (200 mg)	Ph1-2	EMD (≥2 cm)	19/117	52.6%	70.9% (all)	NRep	NR (all)	NRep	31.9 mo (all)	14.3 mo	[71]
**GPRC5D Bispecific Antibody**												
MonumenTAL-1	Talquetamab (0.4 mg/kg QW)	Ph1-2	EMD	33/143	48%	82% (no-EMD)	4.6 mo	9.2 mo (no-EMD)	NRep	Median: NRep; 12-mo: 76% (no-EMD)	25.6 mo	[80]
MonumenTAL-1	Talquetamab (0.8 mg/Kg Q2W)	Ph1-2	EMD	41/154	41%	80% (no-EMD)	3.4 mo	16.9 mo (no-EMD)	NRep	Median: NRep; 12-mo: 77% (no-EMD)	19.4 mo	[80]
Afrough et al.	Talquetamab	RWE	EMD	97/360	68%	65% (no STP)	4.3 mo	7.8 mo (no STP)	10.3 mo	NR (no STP)	12.8 mo	[81]
**Dual Bispecific Combination**												
RedirecTT-1	Talquetamab + Teclistamab (RP2D)	Ph1b	EMD (≥2 cm)	18/44	61%	80% (all)	Median: NRep; 18-mo: 53%	Median: NRep; 18-mo: 70% (all)	NRep	NRep	20.3 mo	[84]
RedirecTT-1	Talquetamab + Teclistamab	Ph2	EMD (≥2 cm)	90/90	79%	—	Median: 15.4 mo; 12-mo: 61%	—	Median: NR; 12-mo: 74%	—	12.6 mo	[85]
**CAR T-Cell Therapy (BCMA)**												
KarMMa	Ide-cel	Ph2	STP (EMD + PSD)	50/128	70%	73% (all)	NRep	8.8 mo (all)	NRep	19.4 mo (all)	13.3 mo	[90]
KarMMa-3	Ide-cel	Ph3	STP (EMD + PSD)	61/254	55.7%	71.3% (all)	7.2 mo	13.3 mo (all)	NRep	NRep	18.6 mo	[96]
KarMMa-3	SOC *	Ph3	STP (EMD + PSD)	32/132	18.7%	41.7% (all)	2.0 mo	4.4 mo (all)	NRep	NRep	18.6 mo	[96]
CARTITUDE-1	Cilta-cel	Ph1b/2	STP (EMD + PSK)	19/97	100%	97.9% (all)	Median: 13.8 mo; 27-mo: 47.4%	Median: NR (all); 27-mo: 54.9%	Median: NRep; 27-mo: 52%	NR (all); 27-mo: 70.4% (all)	28 mo	[91]
CARTITUDE-4	Cilta-cel	Ph3	STP (EMD + PSK)	44/208 35/211	NRep	84.6% (all)	16.9 mo	NE (all)	NE	NE (all)	33.6 mo	[97,98]
CARTITUDE-4	SOC ^#^	Ph3	STP (EMD + PSK)	35/211	NRep	67.3% (all)	4.7 mo	11.8 mo (all)	19.5 mo	NE (all)	33.6 mo	[97,98]
Zanwar et.al.	Ide-cel	RWE	EMD	84/351	54%	82% (no-EMD)	5.3 mo	11.1 mo (no EMD)	14.8 mo	26.9 mo (no-EMD)	18.2 mo	[94]
Sidana et.al.	Cilta-cel	RWE	EMD	60/236	84%	91% (no-EMD)	NRep	NRep	NRep	NRep	13 mo	[95]
**CAR T-Cell Therapy (GPRC5D)**												
MCARH109	MCARH109	Ph1	EMD	8/17	62.5%	71% (all)	NRep	NRep	NRep	Median: NR (all); 3-yr: 59% (all)	37.0 mo	[101,102]
NCT04674813	Arlo-cel	Ph 1	STP (EMD + PSK)	39/84	86%	87% (all)	NRep	18.3 mo (all)	NRep	Median: NR (all); 1-yr: 90% (all)	NRep	[103]
**Dual-Target CAR T**												
Yao et al.	BCMA/GPRC5D CAR T-cell	Ph1	EMD	9/9	100%	—	Median: NR; 1-yr: 63%	—	Median: NR; 1-yr: 60%	—	6.08 mo	[104]
**CAR T vs. BsAb Comparison**												
Steinhardt et al.	Ide-cel	RWE	EMD	22/80	82%	—	7.6 mo	—	24.6 mo	—	16.5 mo	[100]
Steinhardt et al.	Cilta-cel	RWE	EMD	14/80	100%	—	NR	—	NR	—	12.2 mo	[100]
Steinhardt et al.	Teclistamab	RWE	EMD	16/80	36%	—	4.0 mo	—	6.3 mo	—	6.1 mo	[100]
Steinhardt et al.	Talquetamab	RWE	EMD	28/80	29%	—	2.6 mo	—	13.5 mo	—	8.7 mo	[100]

**Abbreviations**: ADC, antibody–drug conjugate; bsAb, bispecific antibody; cilta-cel, ciltacabtagene autoleucel; EMD, extramedullary disease (bone-independent); PSD, paraskeletal disease (bone-dependent); STP, soft tissue plasmacytoma; ide-cel, idecabtagene vicleucel; cilta-cel, Ciltacabtagene autoleucel; arlo-cel, arlocabtagene autoleucel; RP2D, recommended phase 2 dose; RWE, real-world evidence; SOC, standard of care; mo, months; NE, not evaluable; NR, not reached; NRep, not reported; Ph, phase; PSD, Q2W, every 2 weeks; QW, weekly; Q3W, every 3 weeks. * SOC in KarMMa-3 trial were daratumumab, pomalidomide, and dexamethasone [DPd]; daratumumab, bortezomib, and dexamethasone [DVd]; ixazomib, lenalidomide, and dexamethasone [Ird]; carfilzomib and dexamethasone [Kd]; or elotuzumab, pomalidomide, and dexamethasone [EPd]. ^#^ SOC in CARTITUDE-4 were pomalidomide, bortezomib, and dexamethasone [PVd] or daratumumab, pomalidomide, and dexamethasone [DPd]. ^†^ Comparator definitions vary by data availability: (all) = all enrolled patients including those with EMD; (no-EMD) = patients without extramedullary involvement; (no-STP) = patients without soft tissue plasmacytoma involvement, including both EMD and PSD; (—) = EMD-specific study with no comparator. ^Δ^ References correspond to the numbered reference list in the main manuscript.

### 5.4. Other Considerations

#### 5.4.1. Radiation Therapy as an Adjunct to T-Cell-Redirecting Therapies

Radiation Therapy (RT) has traditionally been used in myeloma for palliation, including pain control, prevention of impending fractures, and management of spinal cord compression. However, emerging evidence suggests a broader role for RT in the era of T-cell-redirecting therapies for EMD, with potential applications in two key clinical settings: bridging therapy and consolidation/salvage treatment.

RT before a phase I BCMA-directed CAR T-cell trial was evaluated for its impact on toxicity, response, and CAR T-cell manufacturing outcomes. Patients received RT either more than 1 year before infusion, within 1 year before infusion, or as bridging therapy between apheresis and infusion, with a high prevalence of EMD among those receiving recent or bridging RT. Although RT within 1-year before apheresis was associated with reduced in vitro CAR T-cell proliferation during manufacturing, in vivo CAR T-cell expansion remained comparable across groups. Notably, bridging RT did not negatively affect response rates or CAR T-related toxicities [105]. In another series of 13 patients, including 5 treated with bridging RT before BCMA CAR T-cell therapy, 4 who received salvage RT after CAR T-cell failure, and 4 who received both approaches, the in-field local control rate was 100% at a median follow-up of 7.3 months. No serious adverse events attributable to RT or worsening of CAR T-related toxicities were observed [106].

Beyond local disease control and reduction in tumor burden during the CAR T-cell manufacturing interval, RT may also enhance systemic antitumor immune responses. A case report described a patient who required urgent high-dose steroids and RT for spinal cord compression between days 6 and 20 following BCMA-directed CAR T-cell therapy. The patient developed CRS-like clinical manifestations with increased inflammatory markers that coincided with the expansion of novel T-cell receptor clones after RT. A significant (>30%) increase in T-cell receptor diversity was observed following RT, suggesting a potential synergistic interaction between radiation and CAR T-cell therapy, resulting in an abscopal-like response mediated by radiation-induced neoantigen release [107]. Preclinical data from lymphoma models further support this concept, demonstrating that low-dose fractionated RT combined with CAR T-cell therapy produces additive antitumor effects at both irradiated and nonirradiated tumor sites through activation of the stimulator of interferon genes (STING) pathway. This process enhances CAR T-cell infiltration, increases cross-presentation of tumor-associated antigens, and promotes the development of a systemic effector T-cell response [108].

Consistent with this concept, the European Myeloma Network practical guidelines note that RT can be safely used as bridging therapy in myeloma, particularly in patients with PSD or EMD [109].

Collectively, these findings support the integration of RT into multimodal treatment strategies for EMD, particularly as bridging therapy before CAR T-cell infusion. Beyond tumor debulking, RT may also help overcome the immunosuppressive EMD microenvironment. Spatial transcriptomic analyses have shown that EMD lesions contain exhausted T cells within the tumor core, while functional effector T cells remain restricted to the tumor margins [48]. In preclinical models, low-dose RT can reverse this immune exclusion by promoting T-cell infiltration [110], upregulation of MHC class I, increasing the number of tumor-infiltrating CD8+ T-cells and antigen-presenting cells [111]. These observations raise the hypothesis that RT may sensitize immune-excluded EMD lesions to T-cell redirecting therapies by converting immunologically “cold” lesions into inflamed, T-cell-permissive microenvironments—a concept that requires prospective validation to define optimal timing, dose, and fractionation.

#### 5.4.2. Immunomodulatory Agents and the Inflammatory Microenvironment

The IMiDs illustrate how inflammation-modifying interventions may influence this biology. Although widely used for their anti-myeloma effects, IMiDs were initially developed as anti-inflammatory agents. Lenalidomide suppresses pro-inflammatory cytokines (IL-12, IL-23, IFN-α) and increases IL-10 production by dendritic cells, thereby reducing inflammatory and myeloma-promoting T-cell cytokines [112]. The CD38 monoclonal antibodies demonstrate anti-inflammatory properties beyond plasma cell depletion, reducing multiple pro-inflammatory cytokines (IL-6, IL-10, IL-17, TNF-α, IFN-γ) as well as type I IFN activity [113,114]. Together, these agents shift the microenvironment from a pro-inflammatory state toward a more immunologically permissive one, potentially reducing tumor-supportive signaling and partially restoring immune competence. This provides a biologic rationale for combining IMiDs and/or CD38 antibodies with T-cell-redirecting therapies, particularly given the profoundly immunosuppressive EMD niche characterized by T-cell exhaustion and impaired effector function (Section 4.2), where modulation of the inflammatory milieu may enhance immune competence.

However, this immunomodulatory effect is not unidirectional; an in vivo study of 17 patients with low burden of disease post-ASCT showed that maintenance lenalidomide increased the number and enhanced the suppressive function of Tregs [115]. An in vitro study of healthy donor blood samples examining three types of human dendritic cells (DCs) showed that lenalidomide decreased the capacity of CD1c+ DCs to induce differentiation of naïve CD4+ T-cells into effector T-cells that produce immune-activating (and myeloma-promoting) cytokines [112]. Another in vivo study demonstrated a counter-regulatory effect of lenalidomide, with simultaneous increases in CD8+ effector T-cells (TCM/TEM) alongside Treg expansion and a novel CD14+CD15+ myeloid population with suppressive function (MDSC subtype), suggesting the immune system counterbalances lenalidomide-induced activation [116]. Collectively, these findings indicate that IMiDs have dual immune effects, supporting the need for optimized combination strategies to maximize anti-tumor activity while limiting counter-regulatory suppression.

In addition, CELMoDs, such as mezigdomide and iberdomide, achieve deeper degradation of Ikaros (IKZF1) and Aiolos (IKZF3) compared with conventional IMiDs. Mechanistically, IKZF1 and IKZF3 drive T-cell exhaustion through epigenetic modifications that upregulate exhaustion-associated genes while suppressing cytokine gene expression [117]. Accordingly, beyond their direct anti-myeloma effects, CELMoDs may reverse T-cell exhaustion and restore effector cytokine production [117,118]. In a murine myeloma model, pretreatment with iberdomide/dexamethasone reshaped the BM T-cell compartment by providing a costimulatory signal that facilitated T-cell trafficking and expansion within the tumor microenvironment [119]. In the phase 1/2 study of mezigdomide plus dexamethasone, the ORR in patients with plasmacytomas (broadly defined to include both EMD and PSD) was 30% compared with 41% in the overall population, although survival outcomes specific to this subgroup were not reported [120]. Several ongoing clinical trials are evaluating CELMoD combinations with BsAb; however, EMD-specific efficacy data remain limited.

Beyond immunomodulatory agents, direct cytokine targeting has also been explored in this setting. Despite the strong biological rationale for targeting the IL-6 axis in this context, clinical trials of the anti-IL-6 monoclonal antibody, siltuximab—alone or with standard therapy—did not show clear benefit [121,122,123,124]. However, these studies predated modern T-cell-directed therapies; whether IL-6 pathway inhibition could act synergistically with CAR T-cell therapy or BsAb remains an open and clinically relevant question.

### 5.5. Clinical Implication

The data reviewed here support several practical conclusions for clinicians managing EMD.

1. Distinguishing EMD from PSD is essential, as they differ in biology, prognosis, and treatment response: PSD arises from direct bone extension with contiguity to the BM niche, whereas EMD reflects hematogenous dissemination with BM-independent growth and a worse prognosis [4]. Clinicians should classify and document these entities separately to guide treatment intensity and prognostic counseling.
2. EMD is an aggressive, treatment-resistant phenotype that warrants early escalation to T-cell-redirecting therapies (CAR T-cell therapy or BsAb), as conventional regimens yield poor outcomes (ORR ~20%; median PFS of 6 months) [65].
3. Combination strategies may enhance T-cell-redirecting therapy efficacy by overcoming the immunosuppressive EMD microenvironment. Combining BsAb or CAR T-cell therapy with agents such as CELMoDs, anti-CD38 antibodies, or other targeted therapies may improve responses, although prospective EMD-specific data remain limited.
4. Given the limited efficacy of standard regimens and the emerging role of novel combination strategies, early referral to tertiary centers with access to clinical trials should be prioritized.

## 6. CNS Myeloma: Focus on CAR T-Cell Therapy and Bispecific Antibodies

### 6.1. Definition and Clinical Context

CNS myeloma (CNS-MM) is defined as myelomatous involvement of any of the following: brain parenchyma, spinal cord soft tissue, leptomeningeal disease, or cerebrospinal fluid (CSF) positivity for clonal plasma cells [125,126]. Skull or vertebral plasmacytomas that cause neurologic compression by mass effect alone, without meningeal infiltration, are not classified as CNS disease [127]. CNS-MM is rare, occurring in approximately 1% of patients, and most cases arise in the relapsed or refractory setting [128,129]. Historically, outcomes have been poor, with median overall survival ranging from approximately 2 months in untreated patients to 4–7 months with conventional therapies [129,130,131].

Diagnosis is based on neuroaxis imaging (contrast-enhanced MRI of the brain and entire spine) and CSF analysis including cytology and flow cytometry [125,130]. Risk factors include high-risk cytogenetics, plasmablastic morphology, circulating plasma cells, elevated LDH, and the presence of other EMD sites [129,130]. In the Greek Myeloma Study Group analysis of 4352 patients, EMD was the strongest predictor of CNS-MM (OR: 6.3, 95% CI: 1.5–26, *p* = 0.01) and the strongest negative prognostic factor for post-CNS-MM survival (HR: 2.9, 95% CI: 1.4–5.4, *p* = 0.005) [129].

While standardized CNS response criteria are lacking, our group recently proposed CNS-specific response definitions incorporating both CSF and imaging findings [126]. Complete CNS response was defined as clearance of CSF involvement together with resolution of active CNS disease on imaging, excluding minimal residual T2/FLAIR abnormalities. Partial CNS response was defined as clearance of CSF involvement with partial radiographic improvement, whereas non-response/progressive disease was defined as persistence or progression of radiographic and/or CSF abnormalities.

### 6.2. The Blood–Brain Barrier in CNS-MM

The blood–brain barrier (BBB) limits CNS exposure to most systemic anti-myeloma agents and is what makes CNS-MM pharmacologically distinct from other extramedullary sites [127]. Bortezomib poorly crosses the BBB at therapeutic doses [132], with corresponding lack of clinical activity reported in CNS-MM [133]; other proteasome inhibitors are similarly limited. Daratumumab penetrates the CSF only at low levels comparable to passive IgG diffusion, with clinical activity in CNS-MM reported in case reports [134]. Among conventional agents, IMiDs achieve measurable CSF concentrations after oral administration [135], and a Japanese nationwide survey identified lenalidomide treatment as independently associated with longer OS in CNS-MM (HR: 0.27, *p* = 0.003) alongside intrathecal chemotherapy and radiation [136].

CAR T-cells and BsAb face fundamentally different pharmacologic constraints in the CNS. CAR T-cells are living cellular products that traffic actively across endothelial barriers and expand at sites of antigen, with measurable CSF expansion documented after BCMA CAR T-cell infusion in CNS-MM [137]. BsAb, by contrast, are large IgG-based proteins that depend on passive diffusion across the BBB, which is often disrupted in CNS-MM by the disease itself, prior radiation, or the inflammation that accompanies T-cell engagement. Patients with CNS involvement were excluded from the pivotal trials of all currently approved CAR T-cell and BsAb products, leaving the evidence base for these agents in CNS-MM dependent on retrospective analyses [126,138].

### 6.3. Multimodality Therapy: Radiation and Intrathecal Chemotherapy

Multimodality therapy combining radiation and systemic treatment is the foundation of CNS-MM management [127]. Focal radiation can rapidly stabilize or reverse focal neurologic deficits; whole-brain radiotherapy is an option for poorly localized or cranial nerve disease, and craniospinal irradiation can be utilized as a bridge to consolidative therapy such as CAR T-cell infusion in patients with controlled systemic disease [127]. Even with multimodality radiation, outcomes remain poor; in a series of 45 CNS-MM patients treated with radiation across heterogeneous systemic regimens, median OS was 3.7 months overall and 7.3 months for those achieving complete response [131].

There is limited data on whether myeloma cells are sensitive to intrathecal methotrexate or cytarabine. However, their use could be considered as part of multimodal care in patients with positive CSF for malignant plasma cells [127,139]. In a small retrospective series of 17 CNS-MM patients, those who received intrathecal chemotherapy had significantly longer OS than those who did not (20 vs. 2 months, *p* = 0.02) [140].

### 6.4. CAR T-Cell Therapy in CNS Myeloma

The earliest evidence for CAR T-cell activity in CNS-MM came from a four-patient case series treated with BCMA-directed CAR T, in which three patients achieved complete response, and one achieved partial response; CRS was grade 1–2 in all cases with no ICANS, but durability was limited, with relapses at 81–287 days [141].

A larger multicenter retrospective analysis was subsequently conducted of 10 CNS-MM patients treated with BCMA-directed CAR T (six ide-cel, four cilta-cel) across five US academic centers [138]. The cohort was heavily pretreated, and 60% had concurrent non-CNS EMD. All patients had MRI evidence of CNS involvement, 40% had CSF positivity, and 70% received CNS-directed therapy (predominantly radiation) before CAR T-cell infusion in addition to systemic bridging. Outcomes were notably better than historical reports. The systemic overall response rate was 80% (≥VGPR 70%), and CNS response rate was 100% by day 90. With a median follow-up of 381 days, median PFS was 6.3 months, and median OS was 13.3 months. CRS was grade 1–2 in 80% of patients with no grade ≥3 events; ICANS was grade 1 in 20% and grade 3 in 10%, with no grade 4 ICANS or movement and neurocognitive toxicity (MNT). Best outcomes occurred in patients who responded to bridging therapy, suggesting that pre-CAR T-cell disease control is key for improved outcomes.

### 6.5. Bispecific Antibodies in CNS-MM

The first reported series of BsAb in CNS-MM included nine patients treated predominantly with talquetamab (*n* = 8) across three US centers. Of the nine patients, six were evaluable; all achieved a CNS response and a systemic ≥VGPR, with CRS in 44% (all grade 1–2) and no ICANS [142]. In a larger study by the US Multiple Myeloma Immunotherapy Consortium, 24 patients with CNS-MM were treated with BsAb across 13 institutions [126]. Teclistamab was used in 33%, elranatamab in 17%, and talquetamab in 50%. CNS involvement included brain/cranial nerves (42%), spinal cord (13%), or both (46%). In this heavily pre-treated population, the systemic ORR was 63% (≥VGPR 37%), and the CNS response rate was 58% (complete CNS response 37%, partial 21%). Median duration of CNS response was not reached, and 66% of patients were free of CNS progression or death at one year. Median PFS was 5.0 months (95% CI: 2.5-NR) and median OS was 12.2 months (95% CI: 7.7-NR) at a median follow-up of 11 months.

The safety profile was acceptable. No grade 3–4 CRS was observed. Grade 3 ICANS occurred in 4% (*n* = 1). One patient had delayed parkinsonism, and one had delayed leukoencephalopathy. Infections were common (67% overall, 42% severe), with one infection-related death, and 58% of patients received IVIG. Survival did not differ significantly between BCMA-directed bispecifics and talquetamab, although a non-significant trend favoring the BCMA agents was observed (*p* = 0.11 for PFS; *p* = 0.37 for OS). Like the CAR T-cell studies, most patients (83%) received CNS-directed therapy, including 50% who underwent radiotherapy either alone or in combination with other modalities.

### 6.6. Comparative Outcomes

With the caveat that cross-study comparisons should be interpreted cautiously, the available data suggest meaningful improvements with CAR T-cells and BsAb compared with historical controls (Table 2). Notably, their use was incorporated into a multimodal CNS-directed treatment approach received by the majority of patients in both the CAR T-cell and BsAb studies. CAR T-cell therapy demonstrated notably higher CNS response rates compared to BsAb (100% vs. 58%). This could possibly be attributed to the pharmacologic differences described previously, including the ability of CAR T-cells to traffic to and expand within the CNS, compared with the unproven ability of BsAb to penetrate the CNS in the absence of a compromised BBB. Both modalities had acceptable safety profiles without excess CRS or ICANS compared to the general MM population who receive these therapies.

Given the rarity of CNS MM, the available literature remains limited with respect to the site and extent of CNS involvement, despite extensive multicenter collaborative efforts. Consequently, there are insufficient data to distinguish outcomes between patients with parenchymal CNS myeloma lesions and those with leptomeningeal myelomatous involvement, as well as between patients with bulky CNS plasmacytomas and those with less extensive CNS disease, all of which may represent biologically distinct disease entities. Future studies reporting outcomes according to these patterns of CNS involvement will be important to better define their biology and optimize treatment strategies.

### 6.7. Clinical Implications

The data reviewed here support several practical conclusions for clinicians managing CNS-MM.

1. A history of treated CNS-MM should not, by itself, exclude patients from CAR T-cell or BsAb therapy. Both modalities can be delivered to this population with safety profiles comparable to those in patients without CNS involvement, and patients with controlled or responding CNS disease at the time of T-cell redirection appear to derive the greatest benefit [138].
2. The available evidence, including both major retrospective datasets, supports CNS-directed bridging followed by T-cell redirection as the emerging treatment framework for CNS-MM [126].
3. Given the strong association between non-CNS EMD and CNS involvement (OR 6.3 in the Greek Myeloma Study Group) [129], clinicians should maintain a low threshold for neuroaxis imaging and CSF evaluation in patients with non-CNS EMD, high-risk cytogenetics, plasmablastic morphology, or new neurologic symptoms. Earlier identification of CNS-MM creates a wider window for incorporating multimodality treatment before CAR T-cell therapy or BsAb.

## 7. Future Directions

### 7.1. Improving Outcomes of Soft Tissues (Non-CNS) EMD

Building on the evidence reviewed above, several priorities warrant further investigation to improve outcomes in non-CNS EMD.

- **Multisite and Liquid Biopsy Approaches**

The marked spatial genomic and immune heterogeneity of EMD suggests that single-site biopsies inadequately capture disease complexity. Analogous to metastatic breast cancer practice, where biopsy of both primary and metastatic sites is recommended given receptor discordance [143], integrated sampling of BM and EMD lesions should be pursued to identify actionable alterations and optimize target selection. Liquid biopsy—encompassing circulating tumor DNA and circulating tumor cells—offers a noninvasive means to track clonal evolution and detect extramedullary progression, overcoming the limitations of single-site sampling [144].

- **Overcoming the Immunosuppressive Microenvironment**

Strategies to convert immunologically ‘cold’ EMD lesions into T-cell-permissive microenvironments merit systematic evaluation. Low-dose radiation has been shown to reprogram immunologically cold tumors by promoting T-cell infiltration and enhancing responsiveness to immunotherapy [110] and warrants investigation as a potential sensitizer to T-cell-redirecting therapies in EMD.

- **Novel Therapeutic Targets**

Several targets enriched in EMD warrant prioritization for clinical development, both as single agents and in combination with T-cell-redirecting therapies:

- CD70-directed CAR T-cells and EZH2 inhibitor combinations represent particularly promising avenues [55,59,145].
- *KRAS* mutations in EMD are predominantly non-G12C and only a minority of *BRAF* mutations are V600E, limiting the applicability of mutation-specific inhibitors; broader pan-RAS or MEK-level inhibition may be more universally applicable [36].
- CD24, a marker of drug-resistant and less-differentiated myeloma cells enriched in RRMM and post-BCMA-directed therapy, represents another emerging target of relevance to EMD. Preclinical data [146] demonstrate that dual BCMA/CD24 CAR T-cell constructs outperform monospecific approaches by eliminating dormant resistant cells and restoring macrophage-mediated clearance via CD24–Siglec-10 ‘don’t eat me’ checkpoint blockade, warranting prospective evaluation in the EMD setting.

- **Prospective Trial Design**

Patients with EMD should be prospectively enrolled and analyzed as a distinct subgroup in clinical trials, rather than excluded or pooled with PSD. Dedicated investigation of this biologically high-risk population will be essential to meaningfully improve outcomes.

### 7.2. Improving Outcomes of CNS Myeloma

CNS myeloma remains the most devastating manifestation of EMD, historically associated with dismal survival. However, emerging retrospective data suggest that both BCMA-directed CAR T-cell therapy and BsAb can induce meaningful CNS responses within multimodality treatment strategies. However, several important challenges remain. Despite encouraging initial responses with CAR T-cell therapy and BsAb, the durability of response remains inferior to that observed in EMD and MM overall. Additionally, because CNS myeloma often requires a multimodal treatment approach, close coordination among multiple specialties, including medical oncology, radiation oncology, neurology, and neurosurgery, is essential and frequently necessitates management at a tertiary care center. Efforts to improve outcomes of patients with CNS MM could potentially focus on:

- **Sequencing of Bispecifics and CAR T-Cell Therapy**

The optimal sequencing of BsAb and CAR T-cell therapy in CNS-MM remains undefined. It is worth exploring whether BsAb bridging before CAR T-cell therapy represents an optimal sequencing strategy, particularly given that the best outcome in the BsAb studies described above occurred in a patient who received teclistamab as bridging therapy to CAR T-cell therapy [126].

- **Dual-Antigen Targeting and Combination Therapies**

Antigen escape through loss of BCMA or GPRC5D is a recognized mechanism of relapse following T-cell-redirecting therapy. Whether antigen heterogeneity is even more pronounced in the CNS compartment compared with other EMD sites remains unknown. Dual-targeted BCMA/GPRC5D CAR T-cell constructs have shown early activity in heavily pretreated MM with EMD, while trispecific antibodies are also entering clinical development. Similarly, the talquetamab plus teclistamab combination demonstrated encouraging activity in EMD in the RedirecTT-1 trial, although patients with CNS-MM were excluded. Prospective trials specifically exploring dual targeting of BCMA and GPRC5D in CNS MM are needed. Additionally, combining BsAb with other agents, such as immunomodulatory agents, could be explored.

- **Maintenance Strategies After CAR T-Cell Therapy**

The combination of high initial CNS response rates and relatively short median PFS following CAR T-cell therapy suggests a potential role for maintenance approaches to improve durability. Plausible strategies include BsAb-based, ADC-based, or IMiD-based maintenance, although prospective data are currently lacking.

- **Prospective Trial Design**

The most pressing need in CNS-MM is the development of prospective clinical trials specifically enrolling patients with CNS involvement. Future studies should incorporate standardized CNS-specific response criteria, serial CSF assessments, functional imaging, and correlative pharmacokinetic and antigen-expression analyses to address questions that retrospective datasets cannot resolve.

## 8. Conclusions

T-cell-redirecting therapies are reshaping outcomes in patients with EMD, a biologically high-risk population that until recently had few effective treatment options. CAR T-cell therapy appears to provide the deepest and most durable responses, particularly when incorporated earlier in the disease course, while dual-targeting bispecific combinations have shown especially promising efficacy in this setting.

Nevertheless, durable disease control remains challenging, and EMD continues to confer adverse outcomes even in the immunotherapy era. In CNS myeloma, both CAR T-cell therapy and bispecifics can achieve meaningful responses with acceptable safety profiles, particularly within multimodality approaches that include CNS-directed therapies. CAR T-cell therapy currently demonstrates the highest CNS response rates, potentially reflecting its ability to traffic to and expand within the CNS. Optimal sequencing, maintenance strategies, and dual-antigen targeting in this setting require further investigation. Collectively, the rapid progress of recent years suggests that uniformly poor outcomes in EMD and CNS-MM are no longer inevitable. Prospective clinical trials specifically enrolling patients with EMD and CNS involvement will be essential to further improve long-term outcomes in these historically underserved populations.

## Figures and Tables

**Table 2:** Comparative outcomes of T-cell redirecting therapies in CNS-MM versus traditional approaches.

Outcome Δ	CAR T + CNS-Directed Therapy (*n* = 10)	Bispecifics + CNS-Directed Therapy (*n* = 24)	Traditional Therapies + CNS-Directed Therapy
**Systemic ORR**	80% (≥VGPR 70%)	63% (≥VGPR 37%)	Variable
**CNS Response Rate**	100%	58%	Not systematically reported
**Median PFS**	6.3 months	5.0 months	—
**Median OS**	13.3 months	12.2 months	4–8 months
**Grade ≥ 3 CRS**	0%	0%	N/A
**Grade ≥ 3 ICANS**	10%	4%	N/A

Δ References correspond to the numbered reference list in the main manuscript [126,138,142].

## Data Availability

No new data were created or analyzed in this study. Data sharing is not applicable to this article.
